# Valence Bond Theory—Its Birth, Struggles with Molecular Orbital Theory, Its Present State and Future Prospects

**DOI:** 10.3390/molecules26061624

**Published:** 2021-03-15

**Authors:** Sason Shaik, David Danovich, Philippe C. Hiberty

**Affiliations:** 1 Institute of Chemistry, The Hebrew University of Jerusalem, Jerusalem 9190401, Israel; david.danovich@gmail.com; 2 CNRS, Institut de Chimie Physique UMR8000, Université Paris-Saclay, 91405 Orsay, France

**Keywords:** valence bond, molecular orbital, Lewis, electron-pair bonds, Pauling, Mulliken, Hund, Hückel

## Abstract

This essay describes the successive births of valence bond (VB) theory during 1916–1931. The alternative molecular orbital (MO) theory was born in the late 1920s. The presence of two seemingly different descriptions of molecules by the two theories led to struggles between the main proponents, Linus Pauling and Robert Mulliken, and their supporters. Until the 1950s, VB theory was dominant, and then it was eclipsed by MO theory. The struggles will be discussed, as well as the new dawn of VB theory, and its future.

## 1. Introduction

This essay tells briefly a story of the emerging two major quantum mechanical theories, valence bond (VB) theory and molecular orbital (MO) theory, which look as two different descriptions of the same reality, but are actually not. We discuss the struggles between the two main groups of followers of Pauling and Mulliken, and the ups and downs in the popularity of the two methods among chemists, and then the fall of VB theory only to be revived and to flourish. We end the story with the description of the renaissance in modern VB theory, its current state and its future outlook.

The grassroots of Valence Bond (VB) theory date back to the second decade of the 20th century, when Lewis published his seminal paper entitled, “*The Atom and The Molecule*” [1]. Lewis made use of the discovery of the electron as a fundamental particle of matter, while interjecting his command of chemical facts. These led him to conclude that the most abundant compounds are those which possess an even number of electrons. He therefore formulated the “*quantum unit of chemical bonding*”, an electron pair that glues atoms of most known molecular matter. In so doing, he was brilliantly able to derive electronic structure cartoons that are used to this day and age for teaching and as means of communication among chemists. Lewis further distinguished between shared (covalent), ionic bonds, and polar bonds. He also laid foundations for resonance theory, and used it to explain for the first time color in molecules. He even discussed geometry in terms akin to the valence-shell electron pair repulsion (VSEPR) approach [2].

The contribution of Lewis, and its implementation in 1927–1928 into quantum mechanics by Heitler and London [3,4,5], reached Pauling, who was then in Europe in a mission to learn the new quantum mechanics (and bring it back to the USA). Pauling was excited. He dropped all the previous mechanical models, which he used to teach [6] and began a wide-ranging program of this theory which he called valence bond theory, and which he summarized in his monograph [7]. Pauling’s work translated Lewis’ ideas to quantum mechanics, and the work received very high attention and became extremely popular among chemists.

Molecular orbital (MO) theory was developed at the same time by Hund and Mulliken [8,9,10,11,12,13], and served initially as a conceptual framework in spectroscopy. Somewhat later, Lennard-Jones and Hückel applied MO theory to electronic structures of molecules [14,15,16,17].

Soon enough the two theories and their major proponents started a struggle to dominate the conceptual frame of chemistry. Initially, MO theory was not accepted too easily by chemists, and until the late 1950s, VB theory which used a chemical language was up. Subsequently, MO theory was implemented in useful semi-empirical programs, and was gradually popularized by eloquent proponents like Coulson [18], Dewar [19], and others. These developments and the consequences of the VB–MO struggle, on the reputation of VB theory, determined its gradual downfall. However, with the developments of new conceptual frames and new computational methods during the 1970s onwards, VB theory began to enjoy a renaissance and reoccupy its place alongside MO theory and DFT.

## 2. The Roots and Development of VB Theory

Lewis’ formulation of the nature of the chemical bond is in many ways the precursor of VB theory. Lewis’ paper, “*The Atom and The Molecule*” [1], contains plenty of ideas, some of which were later embedded into VB theory. His electron-pair model and its dynamic nature regarding polarity, was formulated 11 years before the onset of VB theory in physics [2]. Moreover, his work was portable and instrumental for the emergence of the new VB theory of bonding in the hands of Pauling and Slater [2]. It is appropriate, therefore, to begin this section by briefly describing Lewis’ contribution to the nature of the chemical bond.

### 2.1. The Lewis Electronic Cubes and Electron Pair Bonds

While Lewis formulated the electron-pair bonds, he also tried to relate these bonds to the experience of the practicing chemists in the communities of inorganic and organic chemistries [1,20]. In broad terms, the inorganic chemists were observing chemistry of charged species (ions in today’s language), or molecules undergoing dissociation to ions (like acids), while the organic chemists did not observe much of an ionic chemistry, and were interested in the “structure” of their compounds [2,21,22]. The term “structure” was abstract in those early days (mid 19th century), and its representations lumped together groups of atom (which appear in many molecules) and were odd-looking in modern terms (e.g., the initial sausages model of benzene by Kekulé [21,23]). Nevertheless, this was the chemical landscape which Lewis tried to describe in terms of bonds between atoms.

**Figure 1:**
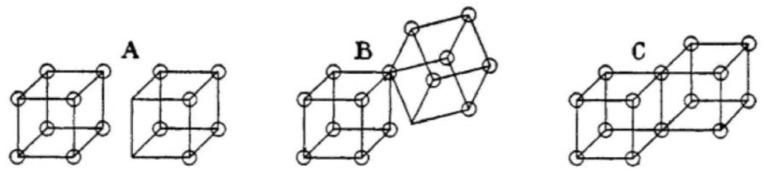
Bonding situations in dihalides, described through the cubic model. Adapted with ACS permission from Ref. [1]. Copyright 1916.

The first model in the key Lewis paper [1] is itself quite cumbersome and is based on his representation of the atom as a cube with a valence-shell that may contain up to eight electrons (later to be called the Octet Rule) placed at the corners of the cube—a model he had developed already in 1902 [24]. Figure 1 describes how Lewis viewed the three putative states of a single bond in the molecule like dihalogen under different conditions using the cubic model. His view of the bond is dynamic and he is aware that a bond can change its character in different environments and depending on the nature of the atoms.

It is seen that the double-cube in **C** represents a “shared electron-pair bond” between the two halogen atoms, which Lewis views as the predominant and characteristic structure of the dihalogens. In addition, both atoms satisfy the octet rule in their valence shell, by sharing an edge which involves *an electron pair*. This shared bond will later be called by Langmuir [25,26] a “covalent bond”. On the other hand, the two cubes in **A** represent an ionic bond, which for Lewis, accounted to a certain extent for the state of I_2_ in liquid iodine. In the middle, in **B**, Lewis describes a case in which “one of the electrons of one ion fits into the outer shell of the second ion [1]”. Reading the Lewis text, it is apparent that he is describing dynamic situations occurring from the covalent form **C** to the ionic one **A**, and passing through **B** which represents a case of intermediate ionicity, i.e., a polar bond (note, that the cartoon **B** maybe viewed also as a one electron bond, though Lewis does not say so).

Within his general idea of dynamic bonds, Lewis discusses this dynamic electronic structure as “tautomerism between polar and non-polar” [20]), and writes: “However, we must assume these forms represent two limiting types, and that the individual molecules range all the way from one limit to the other” [1]. It is clear that Lewis is thinking about a “polar-nonpolar” superposition in bonding, which in a modern dress is Pauling’s covalent–ionic superposition of the electron pair bond [7] (pp. 1–27; 73–100), and in an altered dress will become the mesomerism theory of Ingold [27,28] (pp. 194, 199, 202–207). In the same discussion, Lewis considers intermediate cases in between the extremes—this is a seminal notion of the resonance theory [1,29] (p. 135).

Lewis further uses this mechanism of the dynamic position of the electron pair to allude to heterolysis in solution, when an electron pair moves to one of the atoms. This idea will be fleshed out in the curved arrow invented by Robinson [28] (p. 191) to describe the reaction mechanism, and later by Ingold and Hughes to describe heterolytic processes in organic molecules [28] (pp. 158, 199, 216–220).

**Figure 2:**
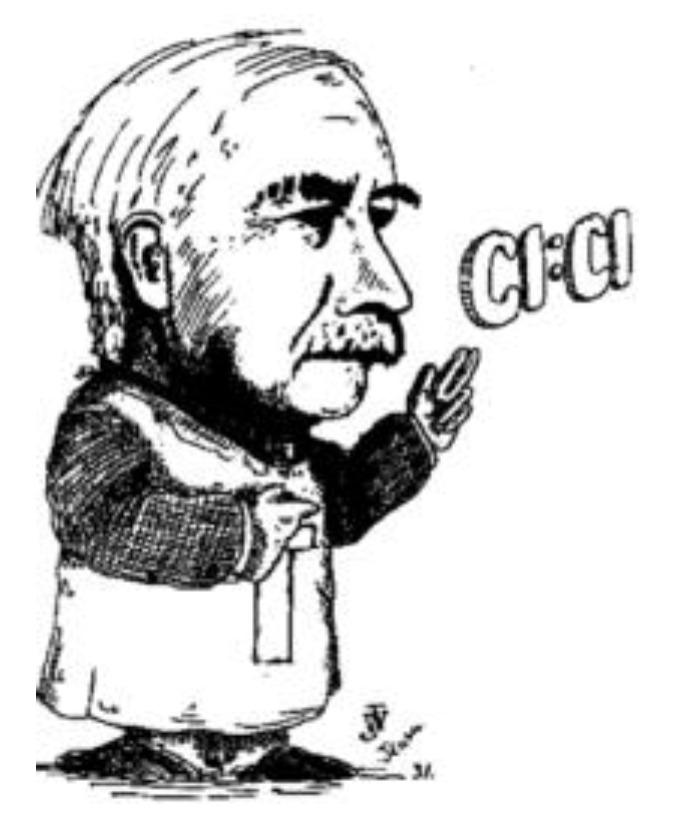
A cartoon of Lewis showing his electron dot model for the electron-pair holding Cl_2_. Adapted by permission of the creator of the cartoon, W.B. Jensen.

Using the cubic model, Lewis tries to describe double and triple bonds between atoms [1]. For a double bond, he describes two cubes sharing a face, such that the two atoms share four electrons, ergo a double bond. However, when Lewis moves on to triple bonds, e.g., in acetylene, he finds the cubic model to be useless. Very soon, Lewis recalls the fact that the helium atom possesses an electron pair (Mosely’s work), and all of a sudden, he changes the cumbersome cubic model in Figure 1, and decides that electron-pair bonding is the fundamental nature of the chemical bond. He then uses the electron-dot structure, as shown in the cartoon in Figure 2.

Subsequently, all the molecular drawings are presented in the electron dot structures [1], whereas in his book, there are also modern representations in which a line connecting the atom replaces the pair of dots [24] (e.g., p. 91). Nevertheless, Lewis goes back to the arrangement of the “*group of eight* (electrons)” which atoms assume in a shared bond [1], and in his Figure 5 (in [1]), he arranges these four pairs in the middle of the cube’s edges in a tetrahedral fashion. As such, he makes a connection to the organic chemists who have been using tetrahedral models for carbon atoms in molecules (e.g., Kekulé in his lectures and Van’t Hoff in his landmark contribution to 3D structure [21]). He further shows that “*two tetrahedra, attached by one, two or three centers of each, represents, respectively the single the double and the triple bond*”. His paper is amazing in this respect since it reads like a stream—a symphony—of thoughts and ideas [1], very different to contemporary papers.

Lewis is aware that others, like Abegg, Kossell, Stark, Thomson and Parson, may have priority claims on various aspects of his theory [30]. He therefore emphasizes that he came up with this idea in 1902, and writes [1]: “*The date of origin [1902] of this theory is mentioned … because the fact that similar theories have been developed independently adds to the probability that all possess some characteristics of fundamental reality*”.

Indeed, as with any great concept, Lewis may have not been the only one to come up with the ideas of electron-pair bonding, or the octet rule (see Box 1). Furthermore, Langmuir followed him [25] and articulated his model further than he did, see Box 1. At the same time, Lewis stayed aloof and did not make special efforts to popularize his model within the chemical community, e.g., by giving talks and/or writing more about it. On the other hand, the more articulate Langmuir contributed a great deal to the dissemination of the Lewis model [30]. Despite of all this, and in retrospect, it is clear that the Lewis approach has prevailed over all others, and has become the “bread and butter” of chemical education and communication amongst chemists. Additionally, further, it constitutes the grassroot of VB theory.

Box 1 **Lewis and others.**

Lewis may have been preceded by Stark, Parson, Thomson and Bohr… Being aware of these publications and likely being challenged by colleagues, he made a point to establish priority [1], and he writes on his cubic model: “*A number of years ago, to account for the striking fact … I designed what may be called the theory of the cubical atom. This theory, while it has become familiar to my colleagues, has never been published*…”, and he adds a footnote: “*These figures are taken from a memorandum dated March 28, 1902*…”, and then he explains his reasons for exacting this date: “*The date of origin of this theory is mentioned… because the fact that similar theories have been developed independently adds to the probability that all possess some characteristics of fundamental reality*”.

There exists a very interesting correspondence between Langmuir and Lewis which is a highly recommended reading [30]. In this correspondence, Langmuir mentions the Bohr model of CH4, in which Bohr used four elliptical 2-electron orbits pointing in a tetrahedral arrangement. He further suggests to rename the model as “*the Thomson-Stark-Rutherford-Bohr-Parson-Kossel-Lewis-Langmuir theory*”. Factually, it is known today as the Lewis model.

### 2.2. Heitler, London, Pauling and Slater, and the Development of VB Theory: Heitler and London’s Study and Follow-Ups

The chemical support of Lewis’ idea presented an agenda for research directed at understanding the mechanism whereby an electron pair could constitute a bond. To physicists, this was not obvious that two negatively charged particles could be “paired”. Indeed, electron pairing remained a mystery until 1927 when Heitler and London (H&L) went to Zurich to work with Schrödinger. Schrödinger was not interested in the chemical bond, but they were … In the summer of 1927, H&L published a seminal paper, *Interaction Between Neutral Atoms and Homopolar Binding* [3], in which they showed that the bonding in H_2_ originates in the quantum mechanical “resonance” interaction which transpires as the two electrons are allowed to exchange their positions between the two atoms. This wave function and the notion of resonance were based on the work of Heisenberg [31]. Thus, since electrons are indistinguishable particles, for two-electron systems, with two quantum numbers *n* and *m*, there exist two wave functions which are linear combinations of the two possibilities of arranging these electrons, as shown Equations (1) and (2).

*Ψ*_A_ = (1/√2)[*φ*_n_(1)*φ*_m_(2) + *φ*_n_(2)*φ*_m_(1)] (1)

*Ψ*_Β_ = (1/√2)[*φ*_n_(1)*φ*_m_(2) − *φ*_n_(2)*φ*_m_(1)] (2)

As demonstrated by Heisenberg, the interference (mixing) of [*φ*_n_(1)*φ*_m_(2)] and [*φ*_n_(2)*φ*_m_(1)] led to a new energy term which splits the energy of the two wave functions *Ψ*_A_ and *Ψ*_B_. He called this term “resonance” using a classical analogy of two oscillators that, by virtue of possessing the same frequency, form a resonating situation with characteristic exchange energy.

**Scheme 1:**
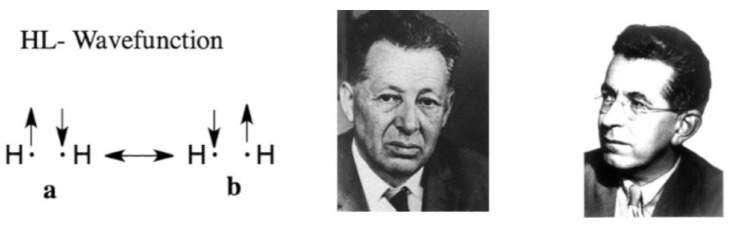
A pictorial representation of the HL covalent form of the H_2_ bond, with spins shown by arrows. On the right side are photos of Heitler and London, from left to right, respectively. Photos of Heitler and London were taken from http://vintage.fh.huji.ac.il/~roib/lecture_notes.htm (accessed on 11 March 2021).

In modern terms and pictorially, the bonding in H_2_ can be accounted for by the wave function drawn in Scheme 1. This wave function is expressed as a superposition of two covalent situations wherein, in form (**a**), one electron has a spin-up (α spin) while the other spin-down (β spin), and vice versa in form (**b**).

Thus, the bonding in H_2_ arises due to the quantum mechanical “resonance” interaction between the two patterns of spin arrangements that are required in order to form a singlet electron pair. This “resonance energy” accounted for about 75% of the total bonding of the molecule in the HL calculations (in modern treatments [32], this is ~90%). As such, the HL wave function in Scheme 1 describes the chemical bonding of H_2_ in a satisfactory manner. This “resonance origin” of the bonding was a remarkable feat of the new quantum theory, since until then, it was not obvious how two neutral species could be at all bonded.

In 1928, London extended the HL wave function and drew the general principles of the covalent bonding in terms of the resonance interaction between the forms that allow interchange of the spin paired electrons between the two atoms [4]. In both treatments [3,4], Heitler and London considered ionic structures for homopolar bonds, but discarded this covalent–ionic mixing as being too small. In 1929, London extended the HL method to a full potential energy surface for the reaction of H + H_2_ [5]. In so doing, he founded a basis for a VB-based approach to chemical reactivity and molecular dynamics (MD). His method created an uninterrupted chain of VB usage from London, through Eyring, M. Polanyi, and all the way to Wyatt, Truhlar, and others.

In essence, the HL theory was a quantum mechanically dressed version of Lewis’ electron-pair theory. Thus, even though Heitler and London did their work independently and perhaps unknowingly of the Lewis model, still, the HL wave function described precisely the shared-pair bond of Lewis. As written above, this issue was forcefully raised by Pauling.

The HL wave function formed the basis for the version of VB theory that became very popular later, and which was behind some of the failings that were to be attributed to VB theory. In 1929, Slater presented his determinant-based method [33], and in 1931, he generalized the HL model to *n*-electrons by expressing the total wave function as a product of *n*/2 bond wave functions of the HL type [34].

In 1932, Rumer [35] showed how to write down all the possible bond pairing schemes for *n*-electrons and avoid linear dependencies among the forms, in order to obtain canonical structures. This level of VB theory, which considers only covalent structures, is referred to as HLVB theory.

Further refinements of VBT [36] between 1928 and 1933 were mostly quantitative, focusing on improvement of the exponents of the atomic orbitals by Wang [37], and on the inclusion of polarization function and ionic terms by Rosen [38] and Weinbaum [39].

**Figure 3:**
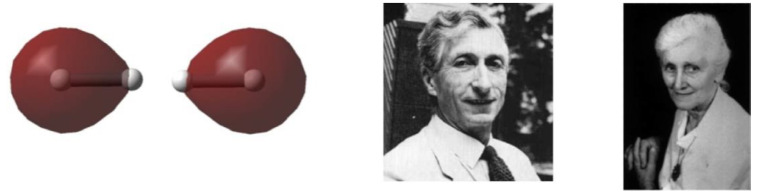
The two Coulson–Fischer AOs, for the H_2_ molecule (using STO-3G for simplicity). On the right are photos of Coulson and Fischer. Coulson’s photo was taken from https://iaqms.org/deceased/coulson.php (accessed on 11 March 2021). Fischer’s photo was taken from http://www.quantum-chemistry-history.com/Fi_Hjal1.htm (accessed on 11 March 2021).

Almost two decades later, in 1949, Coulson and Fischer introduced a new method for calculating covalent bonds. Using H_2_ [40], they showed that by writing the HL wave function and allowing the orbitals to be optimized and delocalized, these atomic orbitals (AOs) developed small delocalization tails on the other hydrogen atom, as shown in Figure 3, and this improves the energy of the molecule. This wave function with AOs having tails on adjacent atoms forms the basis for the modern Generalized VB method (GVB) [41,42,43,44], which will be further discussed later. 

### 2.3. Pauling and Slater

At the time when the HL paper was published, Pauling was in Europe learning the new quantum mechanics (QM) where it originated. He was very excited to see a QM formulation of the Lewis “shared-covalent bond”. He was already aware of and excited about the Lewis paper. In a landmark paper [45], Pauling pointed out that the HL treatments were ”*entirely equivalent to G.N. Lewis’ successful theory of shared electron pair*…”. Thus, although the final formulation of the chemical bond has a physicists’ dress, the origin is clearly the chemical theory of Lewis.

The success of the HL model and its affinity to the Lewis chemical model, posed a great opportunity for Pauling and Slater to construct a general quantum chemical theory for polyatomic molecules. In the same year, 1931, they both published groundbreaking papers in which they developed the notion of hybridization, the covalent–ionic superposition, and the resonating benzene picture [34,46,47,48,49]. In so doing, they formulated thereby a link between the new theory of valence and the nature and 3D structure of key molecular types. Pauling called this new theory, Valence Bond Theory (VBT).

**Scheme 2:**
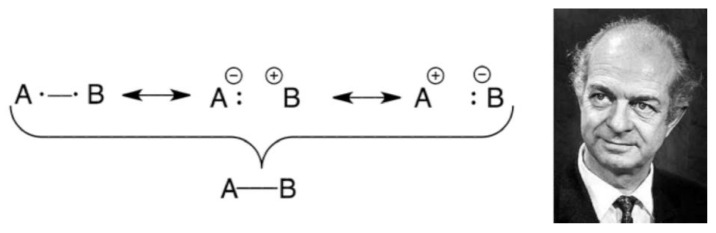
The wave function of a single bond A-B expressed as a resonance hybrid of the covalent HL form and the two possible ionic forms. Shown on the right is the photo of Pauling, taken from https://en.wikipedia.org/wiki/Linus_Pauling (accessed on 11 March 2021).

Especially effective in chemistry were Pauling’s papers. First and foremost, Pauling was a crystallographer, and had a command of the huge structural nuances of molecules. As such, he applied the VB ideas as close as possible to the intuition of chemists. In the first paper [48], Pauling presented the electron pair bond as a superposition of the covalent HL form and the two possible ionic forms of the bond, as shown in Scheme 2, and discussed the transition from a covalent to ionic bonding. In this Scheme, which is analogous to the Lewis Scheme, bonding can change from being mostly covalent through different degrees of polarity, due to mixing of the ionic structures, and all the way to an ionic bond, A^+^:B^−^ (assuming this to be the lower ionic structure):

Later in his book [7] (footnote 13 on p. 73), when he referred to homonuclear bonds, A-A, Pauling stated clearly that the covalent–ionic resonance in such a bond is negligible and assumed to be zero. The covalent–ionic resonance was ascribed only to heteronuclear bonds. As such, Pauling could estimate the covalent bond energy as a geometric mean of the A-A and B-B bond strengths (Equation (3)), while the covalent–ionic resonance was scaled to be proportional to the electronegativity difference (χ_A_ − χ_B_) of the atoms A and B in Equation (4). In so doing, Pauling was able to generate a continuous bond ionicity scale (δ) as a function of the electronegativity difference of the atoms (Equation (5)) [50,51]. The (δ) scale is seen to vary from zero for homonuclear bonds continuously to 1 (full ionicity) for A-B bonds with a very large electronegativity difference. We shall come back to this clever scheme and see its shortcomings.

D_AB_(cov) = (D_AA_D_BB_)^1/2^ (3)

RE_cov-ion_ = D_AB_ − D_AB_(cov) = 23(χ_A_ − χ_B_)^2^, (in kcal mol^−1^) (4)

δ = 1 − exp [−0.25(χ_A_ − χ_B_)^2^] (5)

Pauling and Slater subsequently developed the creative notion of hybridization, which forms localized bonds, which “determine” the molecular geometry. The angles of these hybrids follow the observed bond angles. Thus, for example, sp is the hybridization for co-linear bonds, sp^2^ for three trigonal bonds, sp^3^ for tetrahedral bonds_,_ while sp^3^d and sp^3^d^2^ are for bonds directed to the corners of a trigonal bipyramid and octahedron, respectively. As such, these hybridizations allowed a modern discussion of molecular geometries in a variety of molecules, ranging from organic to transition metal compounds. These hybrids have become very efficient chemistry-teaching tools that allow young students to figure out the geometry of molecules, and in some crude way, also their bonding. Again, this clever scheme will be reexamined later, and its shortcomings will be clarified.

**Scheme 3:**
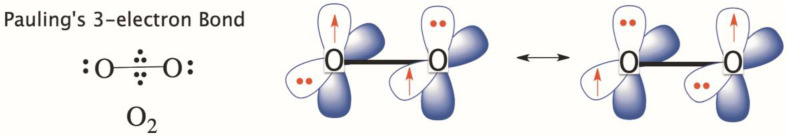
The electronic structure of O_2_ according to Pauling, involving two three-electron π bonds in two perpendicular planes. These are shown in the right drawings, using two resonance structures (1e, 2e ↔ 2e,1e)**.**

In a subsequent paper [49], Pauling addressed bonding in molecules like diborane, and odd-electron bonds as in the ion molecule H_2_^+^, and in dioxygen, O_2_, which Pauling represented as having two three-electron bonds, as shown in Scheme 3. A three-electron bond has two dominant π-resonance structures (1e, 2e ↔ 2e,1e), with two unpaired electrons in the two perpendicular plans of the molecule, having the same spins, and hence, the molecule has a triplet ground state. This Pauling cartoon should surprise any chemist who still holds the opinion that VBT fails and makes a wrong prediction of the electronic structure for the ground state of O_2_. Unfortunately, a close inspection of introductory chemistry textbooks shows that the allegation of failure of VBT for O_2_ is being actively taught.

**Scheme 4:**
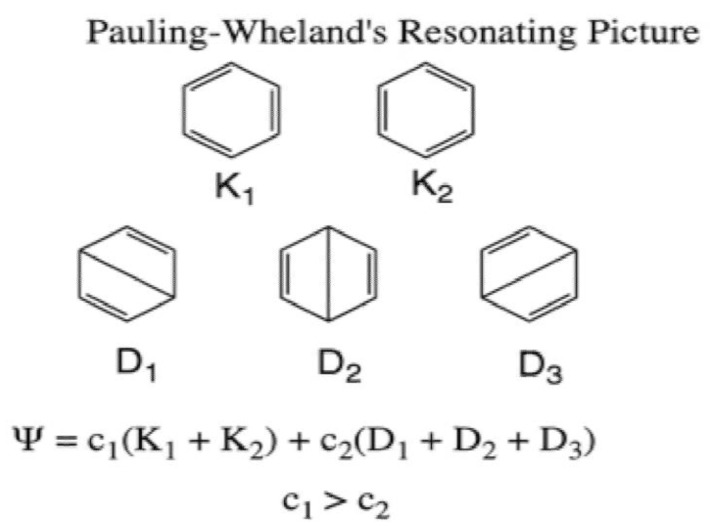
The VB structures (Rumer structures) for describing the π-electronic system of benzene; two Kekulé structures (K_1_ and K_2_) and three Dewar Structures (D_1_–D_3_). K_1_ + K_2_ dominate the wave function.

The description of benzene in terms of a superposition (resonance) of two Kekulé structures appeared for the first time in the work of Slater, as a case belonging to a class of species in which each atom possesses more neighbors than electrons it can share, much like in metals [46]. Two years later, Pauling and Wheland [52] applied the HLVB theory to benzene. As shown in Scheme 4, they used the five Rumer structures of benzene: two Kekulé and three Dewar structures. They further approximated the matrix elements between the structures by retaining only close neighbor resonance interactions.

The corresponding wave function is shown in Scheme 4 below the drawings of the resonance structures, and one can see that the wave function is dominated by the two Kekulé structures, which together form circularly delocalized 6π electrons. This resonance between the two Kekulé structures was calculated to lower the energy of benzene with respect to a single Kekulé structure. Incidentally, the circularly delocalized 6π benzene pretty much resembles Kekulé’s dream [53], which he had one day, in 1861–1862, when he dozed in front of the fire at his home in Ghent. There he saw this molecule as a self-devouring snake writhing on the hexagon periphery. Again, we see Pauling linking his theoretical objects to the chemical graphs of Lewis and his predecessors.

The Pauling–Wheland approach allowed the extension of the treatment to naphthalene and to a great variety of other species. In his book, published for the first time in 1944, Wheland explains the resonance hybrid with the biological analogy of mule = donkey + horse [54]. The pictorial representation of the wave function, the link to Kekulé’s oscillation hypothesis and to Ingold’s mesomerism, which were common knowledge for chemists, made the HLVB representation rather popular among practicing chemists.

While the description of benzene fitted its known excess stability and the ubiquity of its motif in natural products, a similar description of cyclobutadiene, in terms of two HLVB structures, led to a molecule with a more stable π-electronic system than that of benzene. Clearly, this prediction was obviously incorrect and we have to revisit it, when we discuss the MO–VB “wars”.

The above 1931 Pauling papers [48,49] were followed by a stream of five papers, published during 1931–1933 in *Journal of the American Chemical Society*, and entitled “*The Nature of the Chemical Bond”.* This series of papers enabled the description of any bond in any molecule, and culminated in the famous monograph in which all the structural chemistry of the time was treated in terms of the covalent–ionic superposition theory, resonance theory and hybridization theory. The book [7], which was published in 1939, is dedicated to G.N. Lewis, and the 1916 paper of Lewis is the only reference cited in the preface to the first edition. VB theory in Pauling’s view is a quantum chemical version of Lewis’ theory of valence. In Pauling’s work, the long sought for basis for the Allgemeine Chemie (unified chemistry) of Ostwald, the father of physical chemistry, was finally found [29] (p. 135).

## 3. Origins of MO Theory

At the same time that Slater and Pauling were developing their VB theory [36], Mulliken [10,11,12,13] and Hund [8,9] were developing an alternative approach called molecular orbital (MO) theory. The term MO theory (MOT) appears only in 1932, but the roots of the method can be traced back to earlier papers from 1928 [9,11], in which both Hund and Mulliken made spectral and quantum number assignments of electrons in molecules, based on correlation diagrams tracing the energies from separated to united atoms. According to Brush [55,56], Lennard-Jones was the first who has expressed in 1929 a wave function for a molecular orbital wave function, in his treatment of diatomic molecules. In this paper, Lennard-Jones showed with facility that the O_2_ molecule is paramagnetic, and mentions that the HLVB method runs into difficulties with this molecule [14]. The authors of this essay do not really agree with this conclusion since it is very easy to show that the simplest VB theory gets O_2_ correct [22] (pp. 94–97), [57]. Additionally, as we wrote above, there was no obvious reasons for this statement, since VB theory always described this molecule as a diradical with two three-electron bonds (Scheme 3). Nevertheless, this molecule would eventually become a symbol for the alleged failings of VB theory.

**Scheme 5:**
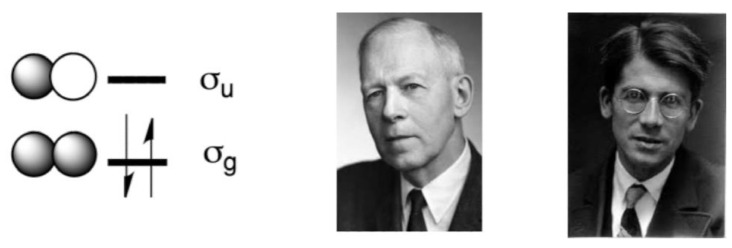
The MO description of the H_2_ molecule. At the simplest level, the bond is an electron pair (with opposite spins) occupying a delocalized MO, σ_g_. On the right side are photos of Mulliken and Hund, respectively; Mulliken’s was taken from https://www.nobelprize.org/prizes/chemistry/1966/mulliken/biographical/ (accessed on 11 March 2021); Hund’s was taken from https://en.wikipedia.org/wiki/Friedrich_Hund (accessed on 11 March 2021).

In MO theory, the electrons in a molecule occupy delocalized orbitals made from linear combination of atomic orbitals. Scheme 5 shows the molecular orbitals of the H_2_ molecule. At the simplest level, the electron pair of H_2_ occupies a delocalized σ_g_ MO. Already with this little molecule, one can see an apparent difference with the HL structures in Scheme 1, and with the more detailed description of an electron pair A-B in Scheme 2. These two cartoons look as though they are describing bonds in alternative universes. Despite the many demonstrations [22] (pp. 40–41) of a subsequent configuration interaction (CI), which includes that the σ_u_^2^ configuration creates a complete equivalence between the VB and MO descriptions of the bond, the pictorial difference has been stamped in the minds of many chemists that VBT and MOT are very different and mutually exclusive theories.

Eventually, it would be the work of Hückel that would usher MO theory into mainstream chemistry. Hückel’s MO theory had initially in the early 1930s a chilly reception [58], but eventually, it gave MOT an impetus and formed a successful and widely applicable tool. In 1930, Hückel used Lennard-Jones’ MO ideas on O_2_, applied it to C=X (X = C, N, O) double bonds and suggested the σ–π separation [15]. With this approximation Hückel ascribed the restricted rotation in ethylene to the π-type orbital.

Using σ–π separability, Hückel turned to solve the electronic structure of benzene [16] by comparing HLVB theory and his new Hückel–MO (HMO) approach. He argued that HMO was preferred since it gave better “quantitative” results (a statement that remains somewhat unclear to the two authors). The π-MO picture, inScheme 6, was quite unique in the sense that it viewed the molecule as a whole, with a σ-frame dressed by π-electrons that occupy three completely delocalized π-orbitals. Comparison of these MO pictures to the five Rumer structures in Pauling’s model (Scheme 4), for benzene, underscores the feeling that these theories are describing benzene in alternative universes.

The HMO picture also allowed Hückel to understand the special stability of benzene. Thus, the molecule was found to have a closed-shell π-component and its energy was calculated to be lower relative to that of three isolated π-bonds as in ethylene. In the same paper, Hückel treated the ion molecules of C_5_H_5_ and C_7_H_7_ as well as the molecules C_4_H_4_ (CBD) and C_8_H_8_ (COT). This enabled him to comprehend the special stability of molecules with six π-electrons, and why molecules like COT or CBD either did not possess this stability (i.e., COT) or had not yet been made (i.e., CBD). Already in this paper and in a subsequent one [17], Hückel laid the foundations for what will become later known as the “Hückel-Rule”, regarding the special stability of “aromatic” molecules with 4*n*+2 π-electrons [55]. This rule, its extension to “antiaromaticity” (4*n* electrons), and its experimental articulation by organic chemists in the 1950–1970s will constitute a major cause for the acceptance of MO theory and the rejection of VB theory [55].

## 4. The MO–VB “Wars”

With these two seemingly different treatments of benzene, the chemical community was faced with two alternative descriptions of one of its molecular icons, and this began the VB–MO rivalry that seems to accompany chemistry to the 21st Century [59]. This rivalry involved most of the prominent chemists of these times (to mention but a few names, Mulliken, Pauling, Hückel, J. Mayer, Robinson, Lapworth, Ingold, Sidgwick, Lucas, Bartlett, Dewar, Longuet-Higgins, Coulson, Roberts, Winstein, Brown, etc. and so forth). A detailed and interesting account of the nature of this rivalry and the major players can be found in the treatment by Brush [55,56].

Interestingly, already back in the 1930s, Slater [47] and van Vleck and Sherman [60] stated that since the two methods ultimately converge, it is senseless to quibble on the issue of which one is better. Unfortunately, however, this more rational attitude does not seem to have made much of an impression on this religious war-like rivalry. This rivalry persisted many years afterwards, even though Hiberty and Ohanessian [61] showed that applying the MO → VB expansion method [62] to the MO-CI wave function of benzene gave rise to the full VB wave function strictly identical to the directly calculated VB wave function [63], and proved the identity of the two methods.

Pauling and Mulliken were the leading figures in this duality that has become sort of a “never ending rivalry” in the generations to come [59]. At times, this rivalry seemed to be personal and even bitter, so much so that in one of the reports of the Löwdin Summer Schools in Vålådalen 1958, the writer (still a student then who did not reveal his/her name) of the report described the relationship between these two great scientists by the orthogonality symbol: <Mulliken|Pauling> = 0.

For a while, the tide was in favor of VBT, because Pauling was very eloquent and persuasive, and because VBT is a chemical language, and hence, it was easier for chemists to grasp it. Furthermore, the condensation of VBT to resonance theory by Pauling made the method so easy to use, and this enhanced its huge popularity. However, this was temporary … The struggle between the Pauling camp and Mulliken’s growing group of followers started to shift in favor of MO theory by the late 1950s onwards, when successful semi-empirical methods [55,56] started to be implemented and could be widely used, e.g., [64,65]. Furthermore, MOT started to have its own eloquent proponents, like Coulson, Dewar, Longuet-Higgins, Hoffmann, etc. The Pauling–Mulliken rivalry, and the avoidance of Pauling to include in his book even a single MO diagram, had its share in the eventual branding of VB theory as a failed theory among the growing number of supporters of the MO approach [59].

However, other major factors combined to make this happen: the fast development of efficient molecular orbital (MO)-based software (the GAUSSIAN suit of programs [66] and others), the synthesis of aromatic and antiaromatic molecules (a dichotomy that initially evaded VB theory), and the formulation of attractive qualitative concepts, like Walsh diagrams, Fukui’s frontier molecular orbital theory, the Woodward–Hoffmann rules of conservation of orbital symmetry [67], and the synthesis of molecules like ferrocene and the elegant interpretation of its unusual bonding by MO theory [59]. The fact that MOT described excited states pictorially and simply, with excitations from bonding to antibonding MOs made it attractive to spectroscopists. Finally, the entrance of density functional theory into chemistry and its formulation in terms of Kohn–Sham MOs [68], which looked like simple Extended Hückel MOs, thus developing the same MO interaction diagram types [69], which made MO theory attractive to chemists [67]. At the same time, VB theory seemed to have stagnated conceptually, and its implementation into an efficient computer code proved to be less successful than that of MO theory. The theory (VBT) ceased to guide chemists to new experiments, though it remained the lingua franca of chemists. However, conceptually, it was cast aside and branded with mythical failures [22] (Chapters 1 and 5).

## 5. Hybridization Is Being Called into Doubt

Ever since hybridization was introduced by Pauling [48] and Slater [46] in 1931, the idea proved extremely insightful and has been extensively used by chemists throughout decades of sustained applications and teaching. Three main categories of hybrid atomic orbitals (HAOs) were defined: the tetrahedral HAOs directed to the corners of a regular tetrahedron, the trigonal ones, lying in the same plane with angles of 120° between them, and the linear hybrids with an angle of 180°. It is well known that the above three hybridization types take place in the prototypical molecules CH_4_, BH_3_ and BeH_2_, respectively, and account faithfully for the bond angles in these systems. More generally, the HAOs show remarkable portability from one molecule to many others. This portability of HAOs in organic molecules is not restricted to bond angles but it also applies to bonding energies, bond lengths and force constants, as exemplified in, e.g., alkanes whose C-H bonds display practically identical properties, different from those of alkenes and alkynes.

Despite its popularity, the hybridization concept has often been criticized, partly for its supposed inability to account for the photoelectron spectroscopy of, e.g., CH_4_ and H_2_O (we will return to this point below), but not only this. The hybridization model was also deemed by some to be useless, and inappropriate for the description of electron density within a molecule [70]. More recently, the sheer legitimacy of hybridization was denied by Brion, who wrote that: “*hybrid orbitals were simply chosen initially by Pauling, and later on implicitly by others, so as to correspond to the supposed localized electron pair chemical bonds, as defined in the classical, pre-quantum G.N. Lewis, purely empirical, localized view of the behavior of electrons […] In contrast, canonical molecular orbitals (CMOs) result directly from the Hartree–Fock procedures without any such additional assumptions*“ [71].

Owing to the diversity of rigorous ab initio calculations, there are several levels of answers to the above negation of HAOs. At the Hartree–Fock level, a well-known property of the single-determinant wave function is that applying unitary transformations (e.g., rotations) on the CMOs does not change the expectation values (e.g., density, energy, etc.) of the determinant. There is an infinity of such unitary transformations, but one is physically more sensible: it is the one that maximizes the distance between the electron pairs that reside in the orbitals [72,73,74].

Such orbital transformations for methane generate a set of localized molecular or bond orbitals (LMOs/LBOs), transformed into one another by the symmetry operations of the *T*_d_ point group, and each containing a tetrahedral HAO pointing precisely toward one of the hydrogen atoms. Two points are noteworthy: (i) the poly-electronic single-determinant wave function made of LMOs, involving the HAOs, is exactly equivalent to the one made of CMOs. It follows that the two Slater determinants yield the same electronic density, the same energy, and the same molecular properties. Therefore, the above-mentioned statement that HAOs are inappropriate for the description of electronic density is unfounded by theory and is strictly wrong. (ii) The tetrahedral hybridization of methane is uniquely determined and arises from the Hartree–Fock calculation without any a priori assumption, as being entirely based on a physical criterion that is nothing else than the VSEPR [75] principle of minimizing the Pauli repulsions between electron pairs. It follows therefore that, at the Hartree–Fock level, the delocalized CMOs and the localized orbitals in terms of HAOs are equally appropriate for the description of chemical bonding.

Furthermore, hybrid orbitals emerge naturally from higher ab initio levels without any assumptions. The highest level of theory that retains the orbital approximation, with fixed orbital occupancies, and uses a single configuration for expressing the total molecular wave function, is provided by the Spin-Coupled Generalized Valence Bond (SCGVB) method [76]. Relative to the Hartree–Fock level, the SCGVB wave function releases the constraint of double orbital occupancy (the orbitals are free to remain doubly occupied or to split into pairs of singly occupied orbitals), and it also releases the constraint of orthogonality between orbitals.

Penotti et al. have shown, in 1988, that the SCGVB wave function of methane yields four atomic orbitals localized on the hydrogens, and four tetrahedral HAOs each pointing to a hydrogen atomic orbital [77]. Furthermore, these authors took into account all the possible ways to perform spin-coupling, and demonstrated that the perfect-pairing way is by far the major wave function of methane, and each of its HAOs is singlet-coupled with the hydrogen AO toward which it is directed. In addition, the tetrahedral molecular geometry is a global minimum on the potential surface at this level of theory, and was therefore not pre-assumed. As such, the shapes of the HAOs arose uniquely in a variational calculation where all constraints are released and where the only preliminary assumption is that one is dealing with a carbon atom surrounded by four hydrogens. On the other hand, there is a logical sequence of constraints that could be placed on SCGVB wave functions to yield the corresponding Hartree–Fock (HF) wave functions [78]. As expected, the SCGVB wave function lies well below the Hartree–Fock one, by some 41 kcal mol^−1^, and only 7.5 kcal mol^−1^ above the full-valence CASSCF. It follows that, at the highest possible level of single-configuration methods, the description of methane in terms of localized orbitals made of HAOs is not merely just as good as the one in terms of CMOs, but definitely better.

Another key feature of these variational hybrids is their p/s ratios, which reflects the tendency to minimize the costly hybridization [78,79] of the central atom (e.g., more than 90 kcal/mol for an sp^3^ hybridization in CH_4_), and afford at the same time the maximum overlap with the ligand atom (H in CH_4_). As such, these variational hybrids deviate from the ideal Pauling–Slater ratios, and variationally depend on the location of the central atom in the Periodic Table. For example, the perfectly tetrahedral hybrids in BH_4_^–^, CH_4_, and NH_4_^+^ possess p/s ratios of 2.38, 1.76, and 1.39, respectively [79], as calculated by multi-structure VB calculations involving the full space of 1764 covalent and ionic structures. As such, directional hybrids are perfectly correct and perfectly reflecting the geometry of the molecules (e.g., tetrahedral for methane, trigonal for BH_3_ and linear for BeH_2_), but their compositions are determined variationally by the promotion energy of the central atom, which varies with its electronegativity. The hybrids behave physically sensible by all means and measures.

## 6. Conceptual Errors Made during the Early Development of VB Theory

Of course, like any new theory, VBT too made some errors in its initial applications to chemical problems. These were, however, errors due to the need for approximations during the calculations, or simplifications that generate useful models, which could not be tested computationally due to the limitations of VB computations at the time of conception.

### 6.1. Assessment of the Covalent–Ionic Bond Scheme

The very clever scheme of Pauling for describing electron pair bonds, A-B, in Equation (3), is partly based on a wrong assumption. Thus, as we showed using modern VB calculations [32,50,51,80,81], the central assumption for the elegant empirical scheme was that RE_cov-ion_(AA) = RE_cov-ion_(BB) = 0, while this assumption is not too bad for H-H, it is extremely poor for F-F. In this bond and others alike (Cl-Cl, O-O, S-S, etc.), the covalent structure is repulsive and the entire bond energy is contributed by the covalent–ionic resonance energy, RE_cov-ion_. As we showed many a time, this Pauling’s assumption has caused the community to ignore a whole family of bonds, in which the bond energy is dominated by the covalent–ionic energy, so-called the charge-shift bond (CSB) family [32,50,51,80,81].

### 6.2. Missing the Antiaromatic Character of C_4_H_4_

The early HLVB treatment of benzene and cyclobutadiene (CBD) by Pauling and Wheland led to a correct prediction that benzene is stabilized by resonance of the two Kekulé structures. However, the resonance energy for CBD came out larger than that of benzene. This was a problem, because unlike the ubiquity of benzene and its motif in many natural compounds, CBD could be made only after a great synthetic effort and strategies to protect the molecule against its high reactivity. Wheland analyzed the problem [52,54,57,82,83], and reached the conclusion that inclusion of the ionic structures to the HLVB method should give a correct prediction. This was an early hint that the neglect of ionic structures in the early VB developments was responsible for the unawareness of the fundamental difference between the (4*n*+2) and 4*n*-electronic systems. Furthermore, the importance of ionic VB structures in conjugated rings was later quantitatively demonstrated by Tantardini et al. [63], who showed that the summed contributions of the two purely covalent Kekulé structures of benzene amount to only 22% vs. 68% for the ionic structures. Subsequently Shurki et al. demonstrated the key role played by the ionic structures in the aromatic/antiaromatic characters of conjugated rings [84]. She showed elegantly, during her Ph.D. with one of us (SS), that the 4*n*+2/4*n* difference is controlled by symmetry; the di-ionic structures which mixed efficiently with the covalent ones in benzene do not do so in CBD due to symmetry mismatch [84]. Of course, the ionic structures may be included either explicitly in the VB wave function, or implicitly through the definition of formally covalent structures with Coulson–Fischer orbitals (vide supra), as in the GVB method. Thus, the GVB treatment of Goddard and Voter [85] showed that CBD has, indeed, a smaller resonance energy than benzene. This calculation also correctly predicted the singlet nature of the ground state, its tendency to distort to a rectangular geometry, and even the sequence of the excited states.

It is also important to note that Hückel theory made a correct prediction for the wrong reason. Thus, as shown by Wheland, the HMO wave function used by Hückel for the singlet state of CBD is equivalent to a single VB structure with two isolated double bonds [82], which is not the correct wave function for CBD in the square geometry. While this orbital choice gave a wrong description of CBD, it accidentally was on the “side of experimental facts” that the species is highly reactive. Furthermore, in the early 1950s, Craig showed that the mono-determinantal MO theory makes a wrong assignment of the ground state of CBD as the triplet ^3^A_2g_ state [86,87] while HLVB gives the correct ground state, ^1^B_1g_. Therefore, the belief that HMO correctly described CBD whereas VB failed is incorrect. In reality, finding the right answer for CBD requires adding the ionic structures to the HLVB treatment [84], or using Coulson–Fischer AOs [85], whereas the MO treatment requires CI [87].

## 7. Myths about VB Failures

Some of the myths that stuck to VBT are discussed in this section, which shows how some myths that were proven wrong, time and again, nevertheless, have lives of their own.

### 7.1. The O_2_ Myth and Mystery

One of the first myths about VB theory is its alleged “failure” to predict the triplet ground state of O_2_. Lennard-Jones stated in his paper that HLVB theory fails to predict the triplet state of O_2_. It is very clear from Scheme 3 that already early on, Pauling discussed the ground state of O_2_ as a triplet state [49]; so did Heitler and Pöschl in 1934, when they discussed the electronic structures of O_2_ and C_2_ [88]. Later, Wheland wrote the same on page 39 of his book [54]. In the 1970s onwards, Goddard et al. [41], Harcourt [89] (p. 50), and the present two authors [22,57] (pp. 94–97), showed again that VB correctly predicts the ground state of O_2_, not only at the ab initio level but already at a simple qualitative level in terms of β integrals and overlaps between atomic orbitals. Nevertheless, this myth still appears even these days in textbooks and papers, as evidenced by a very recent paper on this issue by Corry and O’Malley who try to dispel this myth [90]. What could be the “key” to the enduring persistence of this myth?

It is true that a naïve application of hybridization and perfect pairing approach (simple Lewis pairing) without consideration of the important effect of four-electron Pauli repulsion, in such a structure, would predict a doubly bonded and closed-shall O_2_, which is in fact related to the ^1^∆_g_ excited state of O_2_. As the two present authors showed [22,57] (pp. 94–97), O_2_ avoids the Pauli repulsion and assumes a triplet ground state which is highly stabilized by resonance in its two three-electron bonds. Similar descriptions were given recently by us [91] and by Borden et al. [92]. It remains, therefore, a mystery how this wrong picture could propagate through decades, despite the many VB treatments which showed that the ground state is a triplet state.

### 7.2. The Myth of the Photoelectron Spectroscopies (PES) of CH_4_ and H_2_O

With the emergence of e,2e spectroscopy [93], an old–new myth is being propagated with an attempt to rule out the legitimacy of localized orbitals. This experimental technique of ionizing molecules by collision with an electron beam, as well as the related photoelectron spectroscopy which uses photons for the ionization, led to the claim that these experimental results provide a proof of the initial occupation of electrons in canonical MOs, which is something that defies the fundamentals of quantum mechanics.

CH_4_ is a classical molecule, which once in a while is pulled out as a proof that electrons reside only in delocalized canonical MOs (CMOs). The argument starts from the description of methane as having four localized bond orbitals (LBOs or LMOs) and it goes on as follows: Since methane has four equivalent LBOs, ergo the molecule should exhibit only a single ionization peak in PES. However, since the PES of methane exhibits two different ionization peaks (corresponding to ^2^*A*_1_ and ^2^*T*_2_ states of the cation or to the orbitals of the neutral), ergo VB theory “fails” to predict the ionization spectrum!

This argument is false for two reasons: firstly, as has been known since the 1930s, the LBOs for methane or any molecule can be obtained by a unitary transformation of the delocalized MOs [73,94]. Thus, both MO and VB descriptions of methane can be cast in terms of LMOs/LBOs. Secondly, in VBT like in any quantum theory, the wave function of the CH_4_^+^ cation must be represented by a symmetry-adapted wave function. As such, if one starts from the LBO/LMO description of methane and ionizes the molecule, the electron *can come out of any one of the LBOs*, which are identical by symmetry [22] (pp. 104–106). Hence, a physically correct representation of the CH_4_^+^ cationic state in VBT is a linear combination of the four cationic configurations, which arise due to electron ejection from each of the four bonds. One can achieve the correct physical description, either by combining the LBOs back to canonical MOs [74], or by taking symmetry-adapted linear combinations of the four VB configurations that correspond to one bond ionization [22] (pp. 104–106), thereby producing the ^2^*A*_1_ and ^2^*T*_2_ states of the cation, ^2^*T*_2_ being a triply degenerate VB state of the cation. Thus, the two ionization peaks in PES of CH_4_ are accountable by use of the LMO or VB frameworks as well as in the CMO starting wave function.

The two experimental ionization peaks of H_2_O have also often been invoked as an argument against VB theory and the hybridization concept, which is used in the classical representation of water, with its lone electron pairs located in two equivalent hybrid orbitals, the so-called “rabbit-ears” [70,95,96]. This latter picture is popular among chemists, as it readily explains, for example, the anomeric effect, or the structure of ice, with each water molecule being the site of four hydrogen bonds from neighboring molecules arranged along tetrahedral directions with respect to the oxygen atoms, etc.

As in the CH_4_ case, the rebuttal of this argument is straightforward: the correct VB representation of ionized H_2_O is a combination of two VB structures, which couple the two rabbit ear hybrids in either a positive or negative fashion, leading to two states, ^2^A_1_ and ^2^B_2_, giving rise to two distinct ionization potentials [22] (p. 79).

Thus, here are two myths that have been dispelled and refuted many a time but survive in modern teaching textbooks and in papers. It seems that some chemists are unable to get used to these two seemingly different but otherwise identical pictures.

## 8. Modern VB Theory

The Renaissance of VB theory is marked by a two-pronged surge of activity: (i) Development of new methods and program packages that enable applications to moderate-sized molecules. These have been recently reviewed in a paper by Chen and Wu [97]. (ii) Creation of general qualitative models based on VB theory.

Some of these developments are discussed below, without pretenses of rendering an exhaustive coverage. We of course apologize for omissions.

### Modern Quantitative VBT Approaches

Sometime in the 1970s, a stream of non-empirical VB methods, which were attended by many applications of rather accurate calculations, began to appear. All these programs divide the orbitals in a molecule into inactive and active sub-spaces, treating the former as a closed shell and the latter by some VB formalism. The programs optimize the orbitals, and the coefficients of the VB structures, but they differ in the manners by which the VB orbitals are defined.

Goddard and co-workers developed the generalized VB (GVB) method [41,42,43,44] that uses semi-localized atomic orbitals (having small delocalization tails), employed originally by Coulson and Fischer for the H_2_ molecule (cf. Figure 3) [40]. The GVB method is incorporated in GAUSSIAN and in most other MO-based packages.

Sometime later, Gerratt, Raimondi and Cooper developed a related VB method called the spin coupled (SC) theory, now called “SCGVB“, which also uses atomic orbitals (AOs) with delocalization tails. This is followed up by configuration interaction using the SCVB method [98,99,100]. These methods are now incorporated in the MOLPRO software.

GVB and SCGVB theories do not employ covalent and ionic structures explicitly. Nevertheless, the delocalization tails of these AOs effectively incorporate all the ionic structures [22] (pp. 40–41) and thereby enable one to express the electronic structures in compact forms based on formally covalent pairing schemes.

Balint-Kurti and Karplus [101] developed a multi-structure VB method that may utilize explicitly covalent and ionic structures with local atomic orbitals. In a later development by van Lenthe and Balint-Kurti [102,103] and by Verbeek and van Lenthe [104,105], the multi-structure method is referred to as a VB self-consistent field (VBSCF) method. In a subsequent development, van Lenthe, Verbeek and co-workers generated the multipurpose VB program called TURTLE [106,107], which has been incorporated into the MO-based package of programs GAMESS-UK.

Matsen [108,109], McWeeny [110], and Zhang and co-workers [111,112] developed spin-free VB approaches based on symmetric group methods. Subsequently, Wu et al. extended the spin-free approach, and produced a general-purpose VB program initially called the XIAMEN-99 package, and more recently named XMVB [113,114]. XMVB is becoming faster and more efficient every year, and its VBSCF routine can include up to 26 orbitals/26 electrons in the VB space [97,115], and can also handle molecules with two transition metals and as many as 10 ligands like CO [116]. The new XMVB versions also have DFVB methods, which combine DFT and VBT [117].

Soon after the XIAMEN-99 package, Li and McWeeny announced their VB2000 software, which is also a general-purpose program, including a variety of methods [118]. Another software of multiconfigurational VB (MCVB), called CRUNCH and based on the symmetric group methods of Young was written by Gallup and co-workers [119,120].

During the early 1990s, Hiberty and co-workers developed the breathing orbital VB (BOVB) method, which also utilizes covalent and ionic structures, but in addition, allows them to have their own unique set of orbitals [121,122,123,124,125]. In this manner, BOVB introduces dynamic correlation to bonding and improves the quantitative results vis à vis VBSCF. The method is now incorporated into the TURTLE and XMVB packages.

XMVB is versatile. Thus, Wu et al. [126] developed a VBCI method, which is akin to BOVB, but can be applied to larger systems, since the method starts from VBSCF and improves the orbitals by excitation to virtual orbitals without changing the number of VB structures. In a more recent work, the same authors coupled VB theory with the solvent model, PCM, and produced the VBPCM program that enables one to study reactions in solution [127]. More recent developments involve coupling simple VBSCF calculations (which are fast) with Monte Carlo Simulations to retrieve missing correlation effects [128]. These VB–Monte Carlo methods project a fruitful future direction for VBT.

Finally, Truhlar and co-workers [129] developed the VB-based multiconfiguration molecular mechanics method (MCMM) to treat dynamic aspects of chemical reactions, while Landis and co-workers [130] introduced the VAL-BOND method that is capable of predicting structures of transition metal complexes using Pauling’s ideas of orbital hybridization. A recent monograph by Landis and Weinhold makes use of VAL-BOND as well as of natural resonance theory to discuss a variety of problems in inorganic and organometallic chemistry [131]. Our monograph on VBT [22] includes a chapter that mentions the main program packages and methods, and outlines their features.

The generation of so many different VB methods has advantages as well as some less productive byproducts. Thus, the advent of a number of good VB programs has caused a surge of applications of VB theory, to problems ranging from bonding in main group elements to transition metals [116], conjugated systems, aromatic and antiaromatic species, all the way to excited states and full pathways of chemical reactions, with moderate to very good accuracies. For example, a recent calculation of the barrier for the identity hydrogen exchange reaction, H + H-H’ → H-H + H’, by Song et al. [132] shows that it is possible to calculate the reaction barrier accurately with just eight classical VB structures. Furthermore, just recently, the BOVB and state-averaged BOVB methods were applied to some notoriously challenging excited states, like the ionic V state of ethylene [133] and the 1^1^B_2_ and 2^1^A_2_ states of ozone and sulfur dioxide [134]. In all cases, the BOVB calculated transition energies from the ground state, with less than ten VB structures, were found to be as accurate as standard MO-CI calculations involving tens of millions of configurations! Thus, in many respects, VB theory is coming of age, with the development of faster, and more accurate ab initio VB methods [125].

The less attractive byproduct of the developments of many alternative VB methods, is that this multiplicity does not converge to a unified VB community, but rather creates segmented cults, e.g., preferring localized AOs, or AOs with delocalization tails, or still orthogonal AOs. These cults often criticize or simply ignore one another, and claim uniqueness and truth for one of the choices rather than finding the common ground that stitches all these choices to a single cultural fabric.

What the late John Pople did for MO theory was to assemble many MO methods, and later also DFT methods, under one roof. The MOT front is united because of the leadership of a methodologist with a great vision. VBT is yearning to reach such a situation, with the community of VB proponents focusing on seeing and understanding the other’s method.

Nevertheless, it is a fact that computational VB theory is coming of age, producing faster and more accurate ab initio VB methods.

## 9. Modern Conceptual Approaches in VBT

A few general qualitative models based on VB theory started to appear in the late 1970s and early 1980s. Among these models we also count semiempirical approaches based, e.g., on the Heisenberg and Hubbard Hamiltonians [135,136,137,138,139,140], as well as Hückeloid VB methods [141,142,143,144], which can handle with clarity ground and excited states of molecules. Methods that map MO-based wave functions to VB wave functions offer a good deal of interpretative insight. Among these mapping procedures, we note the half-determinant method of Hiberty and Leforestier [62], and the method by Karafiloglou [145]. Following Linnet’s reformulation of three-electron bonding in the 1960s [146], Harcourt [89,147] developed a VB model that describes electron-rich bonding in terms of increased valence structures and showed its occurrence in bonds of main elements and transition metals.

A general model for the origins of barriers in chemical reactions was proposed in 1981 by one of the present authors (SS), in a manner that incorporates the role of orbital symmetry [22] (ch. 6), [141,148,149]. Subsequently, in collaboration with Pross [150,151] and Hiberty [22] (ch. 6), [152], the model has been generalized for a variety of reaction mechanisms [149], and used to shed new light on the problems of aromaticity and antiaromaticity in isoelectronic series [153].

The present authors have shown the existence of charge-shift bonding (CSB) in electron pair bonds [32], as well as in odd-electron bonds [91]. In addition, Shaik and Hiberty and co-workers have demonstrated that CSBs form a unique family of bonding with special properties and experimental manifestations. One of these manifestations, in chemical reactivity, leads to quantification of the covalent–ionic resonance energy (RE_CS_) of electron-pair bonds [32].

VBT also led to the formulation of the theory of no-pair ferromagnetic (NPFM) bonding [154], which involve triplet-pair bonds (TPB) that can delocalize and give rise to large clusters of monovalent metals, with maximum spin ^n+1^M_n_. The bonding in such clusters can reach as much as ~20 kcal mol^−1^ per single atom. These magnetic clusters are experimentally observable [155,156].

VB ideas have also contributed to the revival of theories for photochemical reactivity. Early VB calculations by Oosterhoff et al. [157,158] revealed a potentially general mechanism for the course of photochemical reactions. Michl [159] articulated this VB-based mechanism and highlighted the importance of “funnels” as the potential energy features that mediate the excited state species back into the ground state. Subsequently, Robb et al. [160] showed that these “funnels” are conical intersections that can be computed at a high level of sophistication. As we (SS and PCH) showed recently [22] (pp. 157–163), the structure of the conical intersection can be predicted by simple VB arguments from VB diagrams. Similar applications of VB theory to deduce the structure of conical intersections in photoreactions were performed by Shaik and Reddy [161].

VB theory enables a very straightforward account of environmental effects, such as those imparted by solvents and/or protein pockets. A major contribution to the field was made by Warshel who has created his empirical VB (EVB) method, and, by incorporating van der Waals and London interactions by the molecular mechanical (MM) method, created the QM (VB)/MM method for the study of enzymatic reaction mechanisms [162,163,164]. His pioneering work ushered the now emerging QM/MM methodologies for studying enzymatic processes [165]. Hynes et al. have shown how to couple solvent models into VB and create a simple and powerful model for understanding and predicting chemical processes in solution [166]]

One of us has shown how a solvent effect can be incorporated in an effective manner to the reactivity factors that are based on VB diagrams [167,168]. More recently, the VB diagram model was applied to reactions in the presence of external electric fields [169,170,171].

Another area where VBT is making promising strides is the description of excited states with a minimal number of VB structures [133,134,143,172,173].

All in all, VBT is seen to offer a widely applicable framework for thinking and predicting chemical trends. Some of these qualitative models and their predictions have been discussed in a monograph on VBT [22] and in review papers [149,150,151,152]. Quantitatively, VBT is improving very fast and is starting to inspire other methods. Thus, a recent benchmark of modern VB methods by Shurki et al. [174] showed that methods like VBCI, BOVB, and VBPT2 exhibit mean unsigned errors as low as 4.5–1.3 kcal/mol, very close to high-level MO-based treatments.

All this activity makes the Lewis-Pauling legacy alive and well!

## 10. VB-Motivated Approaches

As mentioned above [5], London extended the HL method to full potential energy surfaces, and has created a VB-motivated method for MD in chemical reactions. His method was modified throughout the years and is now called LEPS (after London, Eyring, Polanyi and Saito). It is still very useful for studying the full potential energy surfaces of atom transfer reactions [175,176] e.g., for purposes of dynamics and tunneling studies.

VB-motivated methods also infiltrate DFT in terms of pair-density function approaches. Thus, the recently developed method by Truhlar and Gagliardi of separate-pair and separate-pair density functional theory is akin to SCGVB. It is more compact than analogous CASSCF wave functions and is also highly accurate—more accurate than GVB-PP [177,178,179]. This is a useful import of VB ideas into multi-configurational density functional theory, which undergoes continuing development.

Very fruitful VB-motivated methods are those which analyze the probability density of the molecule, and extract thereby information on Lewis bonds, lone pairs, and even covalent and ionic structures [180,181,182].

## 11. Conclusions

Scientific wars leave behind bitterness and lack of understanding for the other views. In the present case, the MO–VB wars created several myths about VB failures (O_2_, anti-aromaticity, etc.), and about its inefficient capability to compute, bonding, structure and reactivity. We hope that this essay straightens out these impressions and shows the richness and beauty of VB theory. Having parallel universes for understanding chemistry and predicting new trends is a boon!

## Figures and Tables

**Scheme 6:**
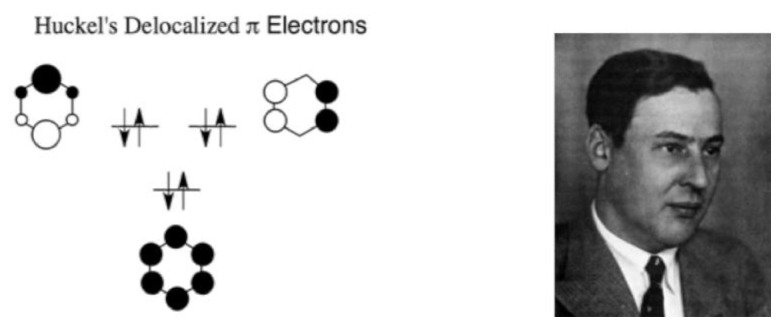
The π-electronic system of benzene by Hückel. On the right is Hückel’s photo, taken from https://en.wikipedia.org/wiki/Erich_H%C3%BCckel (accessed on 11 March 2021).

## Data Availability

Not applicable.
